# Phantom Acupuncture: Dissociating Somatosensory and Cognitive/Affective Components of Acupuncture Stimulation with a Novel Form of Placebo Acupuncture

**DOI:** 10.1371/journal.pone.0104582

**Published:** 2014-08-07

**Authors:** Jeungchan Lee, Vitaly Napadow, Jieun Kim, Seunggi Lee, Woojin Choi, Ted J. Kaptchuk, Kyungmo Park

**Affiliations:** 1 Department of Biomedical Engineering, Kyung Hee University, Yongin, Gyeonggi, South Korea; 2 Martinos Center for Biomedical Imaging, Department of Radiology, Massachusetts General Hospital, Charlestown, Massachusetts, United States of America; 3 Department of Neuropsychiatry, College of Korean Medicine, Sangji University, Wonju, Gangwon, South Korea; 4 Program in Placebo Studies, Beth Israel Deaconess Medical Center, Harvard Medical School, Boston, Massachusetts, United States of America; Brown University, United States of America

## Abstract

In a clinical setting, acupuncture treatment consists of multiple components including somatosensory stimulation, treatment context, and attention to needle-based procedures. In order to dissociate somatosensory versus contextual and attentional aspects of acupuncture, we devised a novel form of placebo acupuncture, a visual manipulation dubbed phantom acupuncture, which reproduces the acupuncture needling ritual without somatosensory tactile stimulation. Subjects (N = 20) received both real (REAL) and phantom (PHNT) acupuncture. Subjects were retrospectively classified into two groups based on PHNT credibility (PHNTc, who found phantom acupuncture credible; and PHNTnc, who did not). Autonomic and psychophysical responses were monitored. We found that PHNT can be delivered in a credible manner. Acupuncture needling, a complex, ritualistic somatosensory intervention, induces sympathetic activation (phasic skin conductance [SC] response), which may be specific to the somatosensory component of acupuncture. In contrast, contextual effects, such as needling credibility, are instead associated with a shift toward relative cardiovagal activation (decreased heart rate) during needling and sympathetic inhibition (decreased SC) and parasympathetic activation (decreased pupil size) following acupuncture needling. Visual stimulation characterizing the needling ritual is an important factor for phasic autonomic responses to acupuncture and may undelie the needling orienting response. Our study suggests that phantom acupuncture can be a viable sham control for acupuncture as it completely excludes the somatosensory component of real needling while maintaining the credibility of the acupuncture treatment context in many subjects.

## Introduction

While acupuncture has been shown to reduce pain in many previous clinical trials, statistically significant differences between real and sham acupuncture have not been consistently demonstrated [1], [2], [3], [4]. This may be due to the fact that sham acupuncture commonly has included a somatosensory or tactile component. In fact, previous studies have not separated the complex acupuncture ritual into its constituent components, which could better determine the specific effects of this therapeutic intervention [1]. In this study, we propose an experimental design that allows for a separation of the acupuncture ritual into a somatosensory and contextual component, with autonomic outflow and psychophysical outcome metrics.

Sham acupuncture, which has been used as a control in many acupuncture studies, has been shown to produce a physiological effect [5], [6], as it affects skin receptors, which are known to be even more dense than muscle and fascial somatosensory and nociceptors [7], [8]. In fact, sham acupuncture can produce similar somatosensory or pain intensity even without skin penetration [9], [10]. Acupuncture is a multi-dimensional intervention, and usage of sham acupuncture techniques as controls in clinical trials would be aided by a better understanding of the different components related to the therapeutic effect of acupuncture [11]. For instance, the tactile component in sham acupuncture is considered essential for credibility of the needling ritual. But this may not be the case. Because tactile stimulation produces a physiological response and may overlap therapeutic components of verum acupuncture, usage of sham acupuncture as a placebo control may be compromised, and there is great need for the development of a credible sham acupuncture procedure that does not include somatosensory (tactile) stimulation [12].

In this study we employed several outcome measures to assess different components of acupuncture. Physiological measures estimated autonomic nervous system (ANS) activity. ANS responses have been reported to have clinical relevance to many disease processes. For example, heart rate changes have been associated with clinical improvements for PTSD [13] and chronic pain [14], and have been linked with memory recall [15], somatosensory processing [16] and emotional memory processing [17]. Moreover, many studies have explored acupuncture’s effects on ANS activity. Yao et al. showed that acupuncture induces a temporary increase in sympathetic tone, followed by a more prolonged depression [18]. Other investigators have also noted increased sympathetic tone during acupuncture stimulation [19] and increased parasympathetic tone after the stimulation [20], [21]. Other studies have linked acupuncture-induced HR decrease, with immune system modulation [22]. However, Lee et al. [23] found no definitive evidence of association between heart rate variability and clinical outcomes, perhaps due to variability in stimulation methods (e.g. needling intensity, duration and frequency), experimental conditions (e.g. measurement timing), or between-subject variability. In fact, individual autonomic response is easily influenced by subtle changes of experimental setup, necessitating a well-controlled design. Our previous study measured concurrent autonomic and brain responses in a neuroimaging study [5]. We showed that, on average, acupuncture produced HR decrease and SCR increase after manual needle stimulation, though individual stimuli could produce both HR increase and decrease. These variable responses were modulated by distinct neural circuitries. In summary, acupuncture induced ANS response may vary based on needling location, needling technique, and needling dose as well as psychological factors and temporal variability.

In this study, we developed a novel form of sham acupuncture which was credible for many subjects, but did not include somatosensory stimulation. This allowed us to dissociate three different components of acupuncture. These included a tactile stimulation-specific component, an attentional shift component (due to visual/somatosensory stimulation), and a cognitive component related to a credibility of the needling procedure. Psychophysical and psychophysiological outcomes were used to dissociate these different components of acupuncture.

## Materials and Methods

All research procedures were approved by the Institutional Review Board (IRB) committee of Sangji University (IRB approval number: SJ 2007-071201), and investigations were conducted in accordance with the principles of the Declaration of Helsinki. All participants in the study provided written informed consent.

### Subjects and Experimental Design

Twenty healthy, right handed female adults (21.8±2.6 years old) participated in both real acupuncture and sham control (phantom acupuncture) sessions in a crossover design. Subjects were recruited via fliers/webpage at the university and its neighborhood. Subjects were screened to exclude any autonomic dysfunction and asked not to take any pharmacological or autonomic modulating substance (e.g. caffeine) prior to testing.

A behavioral training session was completed 1 week prior to either experimental session, during which acupuncture stimulation was applied to the subjects in exactly the same way as during the real acupuncture (REAL) session to record a video clip of acupuncture needling prior to the actual acupuncture sessions. During this session, the acupuncture expectancy questionnaire was completed by study subjects to quantify individual variability in expectancy for acupuncture efficacy [24]. Subjects also completed the Edinburgh Handedness questionnaire [25]. The acupuncturist for this all subsequent sessions was an experienced practitioner (WC) with five years of clinical practice.

**Figure 1:**
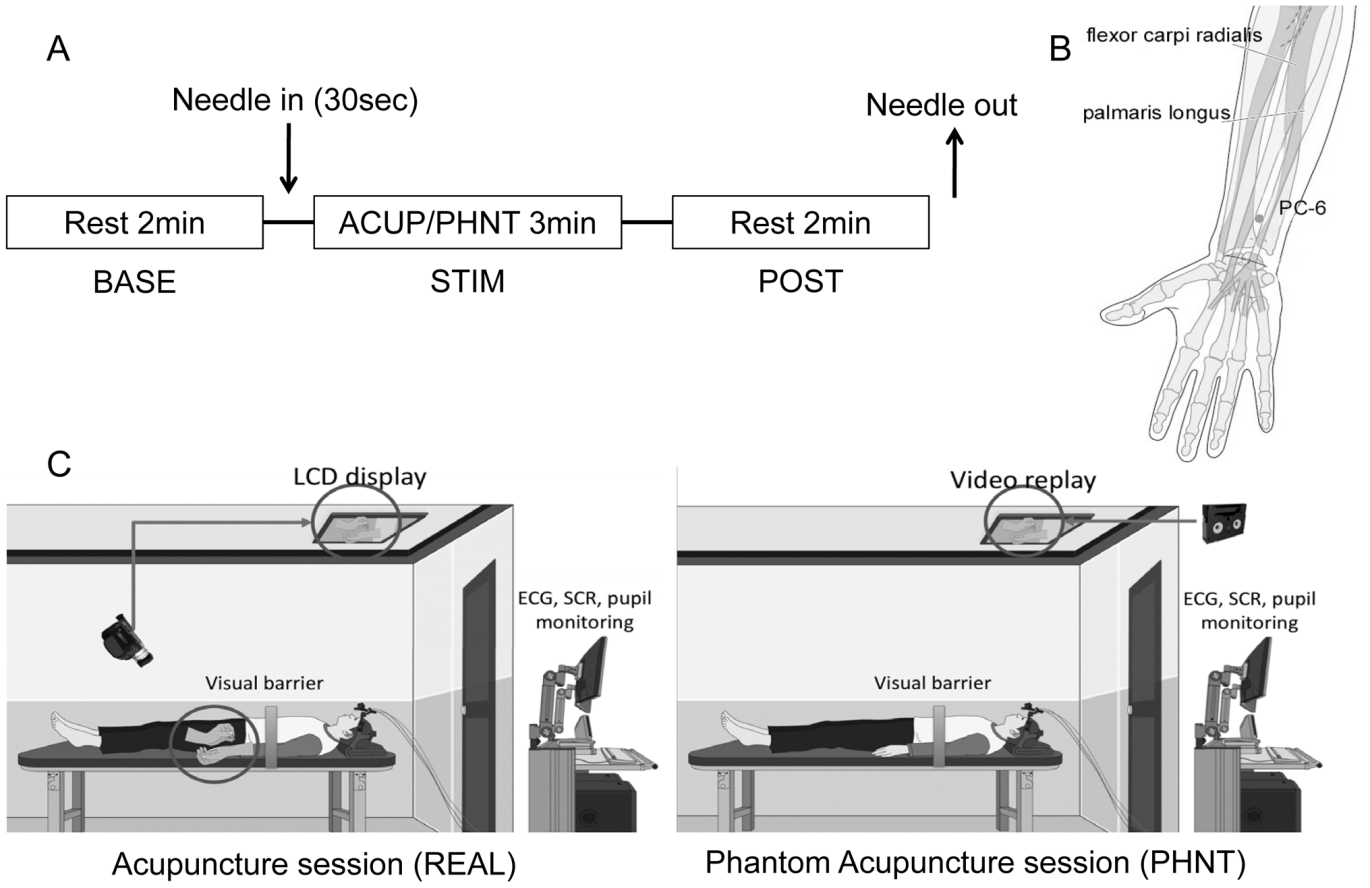
Experimental protocol. **A.** The paradigm consisted of 3-minute event-related stimulation (STIM, 3-second stimulation, ISI mean = 19.5 sec) surrounded by two 2-minute rest sessions (BASE and POST). **B.** Acupuncture stimulation location (PC6). **C.** Experimental setup for REAL and PHNT sessions. n.b. Figure in **B.** was modified from an image in ‘WHO Regional Office for the Western Pacific, 2008, WHO Standard Acupuncture Point Locations in the Western Pacific Region, Manila’.

Real acupuncture (REAL) and phantom acupuncture (PHNT) sessions, separated by at least 40 min, were performed with pseudo-randomized order. For REAL, a 2-minute duration resting baseline (BASE) was followed by needle insertion (completed within 30 seconds, **Figure 1B**). The needle (0.3 mm*30 mm stainless steel needle, Dongbang Co., Korea) was inserted at left acupoint PC6 on the medial side of the right forearm and rotated manually at a rate of ∼2 Hz. This acupoint is 2 cun (approximately 5 cm) proximal to the transverse wrist crease, between the tendons of the palmaris longus and flexor carpi radialis muscles, and is innervated by the median and antebrachial cutaneous nerves. This point is thought to be useful for cardiac conditions, as well as to control nausea and vomiting. It was chosen for this study because clinically, stimulation at this point has been used to modulate autonomic function and is known to induce robust acupuncture sensation [26].

The acupuncturist approached his hand to the acupoint and rotated the needle according to the stimulation timing that was implemented by computer (Psychtoolbox and Matlab, The MathWorks Inc., MA, USA) and relayed to the acupuncturist by auditory signals via headphones. Needle stimulation (STIM) comprised eight stimuli (3-second durations) at pseudo-randomized inter-stimulus interval (μ = 19.5 second) over a duration of 3 minutes. Another 2 minute resting period (POST) followed this STIM period, where the acupuncture needle was retained in the arm (**Figure 1A**). The entire procedure (lasting ∼7.5 minutes) was video-recorded and simultaneously displayed to the subject, who could not see the procedure directly due to a visual barrier.

For PHNT, the acupuncturist did not provide any tactile input to the subjects, and only approached his hand toward the acupoint. However, the video clip of prior acupuncture needling which was recorded at the previous REAL or training session was replayed to the subject, thus creating an illusion of needle insertion and stimulation **(** **Figure 1C** **)**.

In order to perform the needle stimulation at the exact timing according to the experimental protocol, the acupuncturist followed auditory cues. After the stimulation timing signals were sent to the acupuncturist, the approaching time of acupuncturist’s hand to the acupoint (PC6) in the video display were calculated retrospectively. The average time delay from onset of hand motion to reaching the acupoint was 0.77±0.26 second (mean±STD). Thus, actual acupuncture stimulations were applied about 0.8 second after the subject observed visual motion for the acupuncturist’s hand.

Subjects were either acupuncture-naïve (n = 2) or had only a few experiences with acupuncture treatment (n = 18, 6.0±6.7 times, mean±STD), and were informed that there would be two identical experimental acupuncture sessions. Subjects laid supine with their vision of distal body regions blocked by a barrier. They were told to look at the video display projected onto a monitor on the ceiling and were thus prevented from viewing the intervention occurring at the acupuncture point in their periphery **(** **Figure 1C** **)**.

The PHNT session aimed to control for ‘needling credibility,’ but without somatosensory afference. In turn, the REAL session included both the ‘somatosensory stimulation’ as well as the needling credibility inherent to acupuncture.

### Psychophysical Data Collection and Analysis

After each session, subjects were presented with a 10-point VAS and were asked to rate the intensity of different sensations they felt during the STIM period. We used an in-house Korean version of MGH Acupuncture Sensation Scale [27] comprising different “*deqi*” sensations (i.e. aching, soreness, pressure, heaviness, fullness, warmth, cool, numbness, tingling, and dull pain). In order to quantify the total intensity of acupuncture sensation experienced, we used the previously described MASS-Index [27]. This index attempts to balance breadth and depth of sensations as well as the number of different sensations chosen by the subject. The MASS index (MI) and individual sensation intensities were compared between stimulation groups using a paired *t*-test, significant at p<0.05.

### Retrospective Re-classification According to Needling Credibility

**Figure 2:**
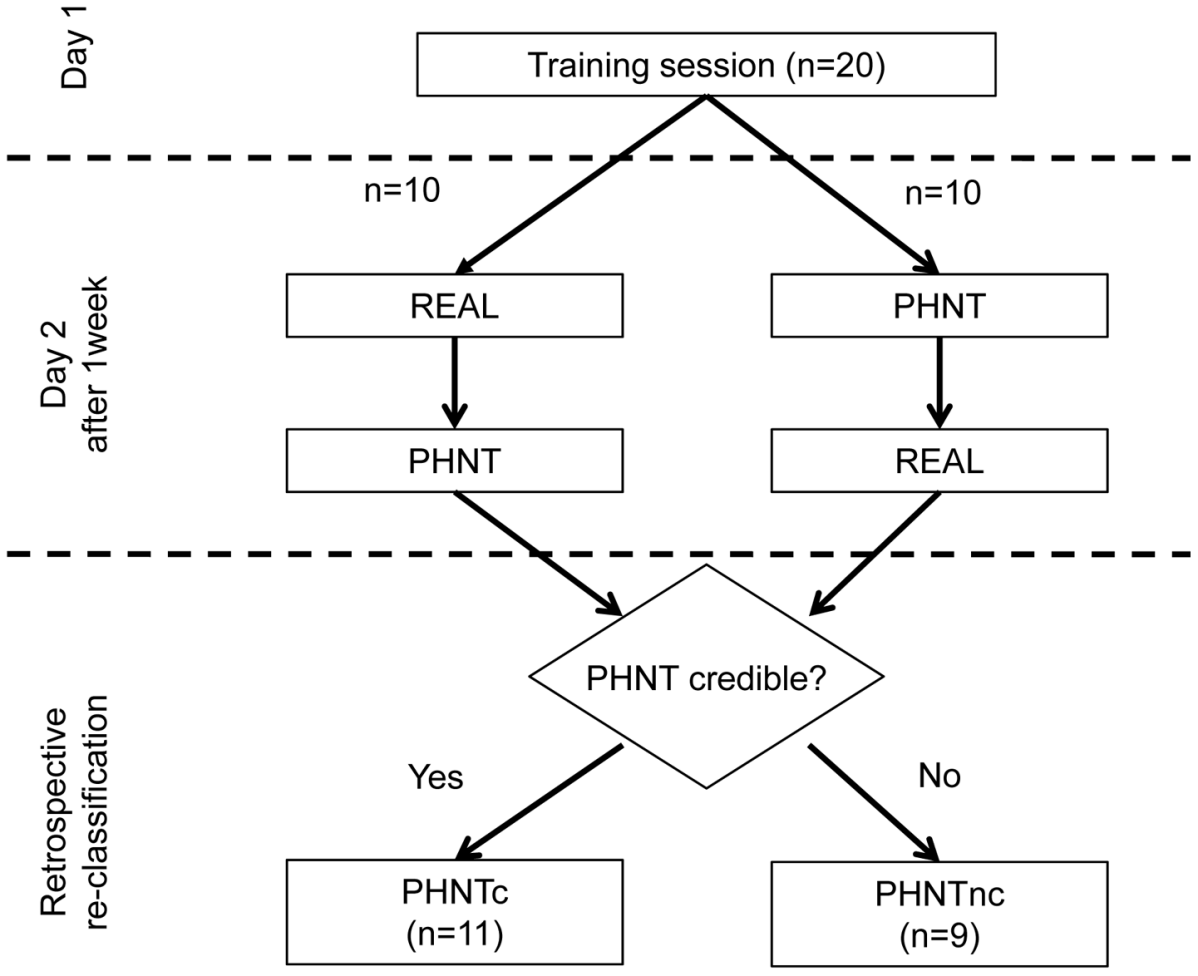
Study flow. Among twenty healthy subjects, ten received real acupuncture (REAL) first, while the rest received phantom acupuncture (PHNT) first, and they were re-classified into phantom credible (PHNTc) and phantom non-credible (PHNTnc) according to the needling credibility in phantom acupuncture (PHNT).

After finishing both REAL and PHNT sessions, subjects were retrospectively separated into phantom credible (PHNTc, high needling credibility, n = 11) and phantom non-credible (PHNTnc, low needling credibility, n = 9) groups using a questionnaire and interview that evaluate the credibility of the procedure – e.g., whether or not they were able to differentiate the difference between real and phantom acupuncture, and if they believed they received real needle acupuncture in both sessions. Four subjects in the PHNTnc subgroup recognized the procedure as placebo when they noticed that the video clip of acupuncture needling was not synchronized with their hand’s spontaneous movement. Five subjects in the PHNTnc subgroup had low credibility because they did not have any acupuncture sensation at the acupoint or surrounding region (**Figure 2**).

### Multi-modal Physiological Data Collection and Analysis

To investigate any autonomic modulation specific to somatosensory afference or needling credibility following acupuncture, we recorded heart rate (HR), skin conductance (SC), and pupil size (PS) throughout the entire session (7.5 min). Subjects rested for at least 10 minutes prior to initiation of data collection. Electrocardiogram and electrical skin conductance were measured using commercial devices (PowerLab/800, ADinstruments, Australia) with a 1 kHz sampling rate. Pupil diameter was measured using a custom constructed pupilometry system that includes an image acquisition system (an IR camera and optical devices attached on a helmet) and analysis software enabling the estimation of precise pupil diameter for every image frame (30 frames/sec) using geometric correction, which compensate the errors induced by lens of the camera and by projection on two-dimensional image plane [28].

ANS outflow metrics (HR, SC, and PS) were computed for estimation of both a tonic response and a phasic event-related response. For the tonic response, the mean HR, SC and PS were calculated for three separate windows: BASE, STIM, and POST. For the phasic responses, the maximum change scores (typically decrease for HR, and increase for SC and PS) were calculated in a 6 second window (0∼6 seconds after each stimulation onset), which was contrast to a baseline window (preceding 5 seconds, accounting for acupuncturist hand motion as previously mentioned; i.e., −5.8 to −0.8 sec, with −0.8 to 0 second excluded due to acupuncturist’s reaction time).

## Results

For the 20 subjects, order of REAL or PHNT session was pseudo-randomized such that 10 received real acupuncture first, while the rest received phantom acupuncture first. There was no significant difference in age (REAL first: 21.1±2.9 years old, PHNT first: 22.5±2.3 years old; mean±STD), handedness (REAL first: 73.4±28.9%, PHNT first: 68.3±54.7%; 100%: right handed, −100%: left handed), positive expectation about acupuncture efficacy (REAL first: 2.4±0.2, PHNT first: 2.3±0.5; out of 1 to 5 range) or state/trait anxiety (STAI-state: REAL first: 27.8±7.5, PHNT first: 26.8±5.6; STAI-trait: REAL first: 33.7±5.2, PHNT first: 31.8±8.3) between the two order groups. From retrospective credibility questionnaires, we classified our subjects into PHNTc (PHNT credible; who reported high needling credibility for PHNT, n = 11) and PHNTnc (PHNT non-credible; who reported low needling credibility, n = 9).

### Needling credibility effects: PHNTc vs PHNTnc

**Figure 3:**
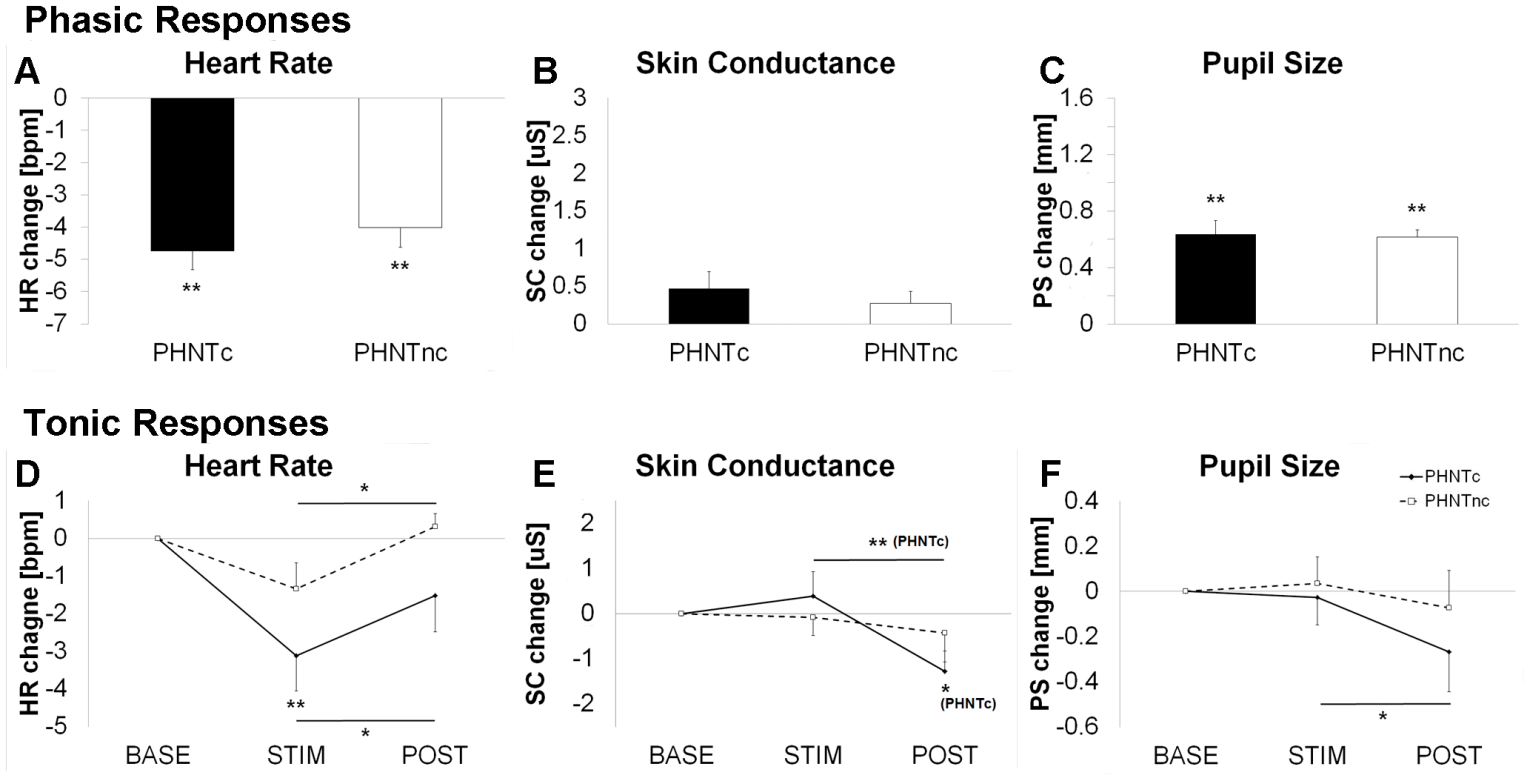
Influence of credibility on autonomic response modulation to phantom acupuncture. Phasic and tonic responses for heart rate (**A** and **D**), skin conductance (**B** and **E**), and pupil size (**C** and **F**) were contrasted between credible (PHNTc) and non-credible (PHNTnc) phantom acupuncture. n.b. *<0.05, **<0.01. Error bars represent standard error of the mean.

For phasic ANS responses, regardless of needling credibility, we noted decreased HR (PHNTc = −4.75±0.58 BPM, P<0.001; PHNTnc = −4.01±0.62 BPM, P<0.001, mean±SEM) and increased PS (PHNTc = 0.64±0.10 mm, P<0.001; PHNTnc = 0.62±0.05 mm, P<0.001) in response to visual stimulation. We did not note a phasic SC response. No significant differences were noted between PHNTc and PHNTnc in terms of phasic ANS response (**Figure 3A–C**).

For tonic ANS responses, we noted significant decrease in tonic HR for PHNTc (Δ = −3.12±0.94 BPM, P<0.01) but not for PHNTnc (Δ = −1.33±0.68 BPM, P = 0.09) during STIM (compared to BASE). During POST, the tonic HR rebounded back to BASE levels. For SC and PS, only PHNTc showed significant tonic decreases during POST compared to STIM (SC response: Δ = −1.27±0.45 µS, P<0.001; PS response: Δ = −0.27±0.18 mm, P<0.05) (**Figure 3D–F**).

**Figure 4:**
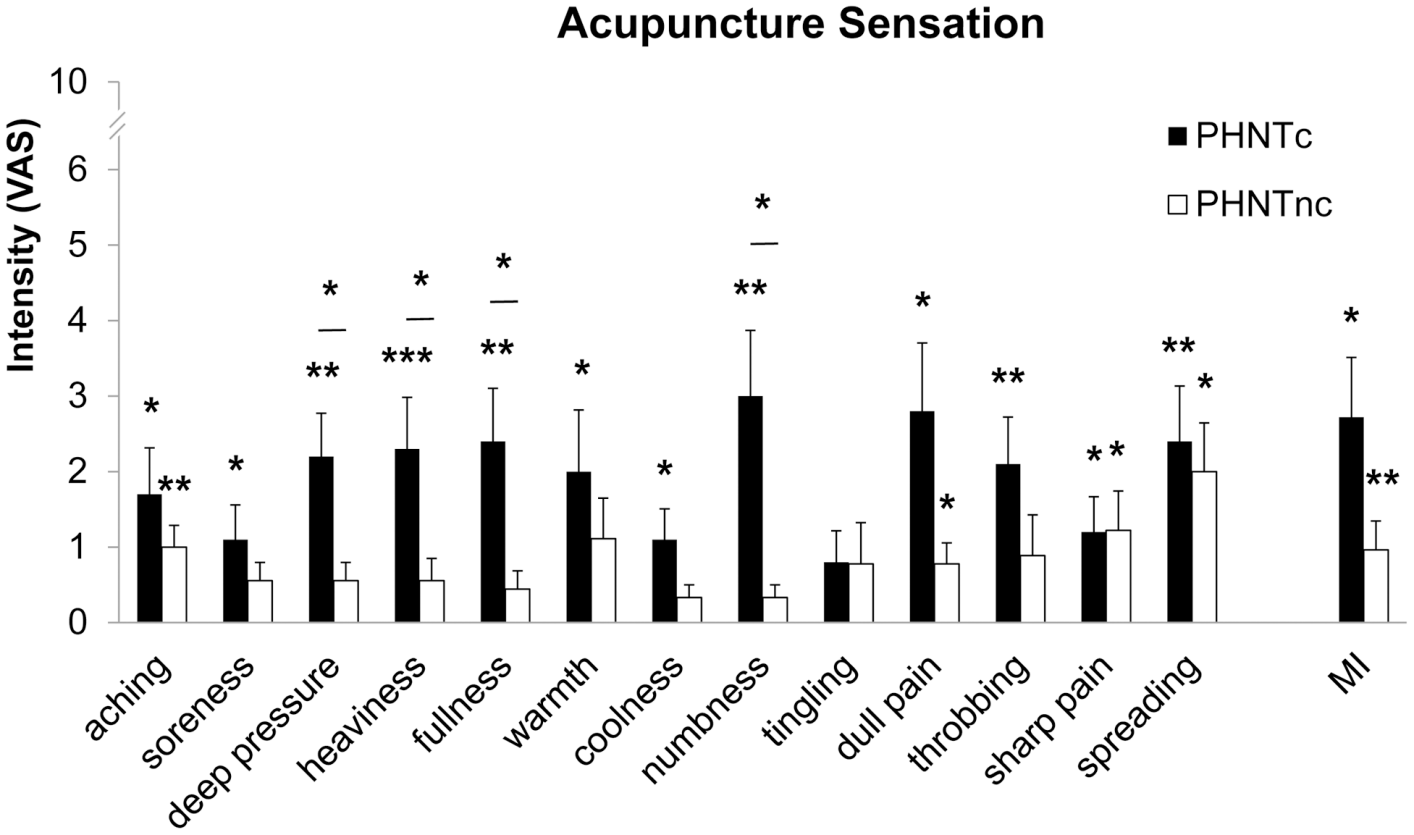
Influence of credibility on acupuncture sensations to phantom acupuncture. PHNTc reported significantly greater sensation intensity for numbness and dull pain (i.e. *deqi* sensations). n.b. *<0.05, **<0.01, ***<0.001. Error bars represent standard error of the mean.

Interestingly, following PHNTc, subjects reported many different acupuncture sensations (e.g. aching = 1.7±0.6, P<0.05; soreness = 1.1±0.5, P<0.05; deep pressure = 2.2±0.6, P<0.01; heaviness = 2.3±0.7, P<0.001; fullness = 2.4±0.7, P<0.01; warmth = 2.0±0.8, P<0.05; coolness = 1.1±0.4, P<0.05; numbness = 3.0±0.9, P<0.01; dull pain = 2.8±0.9, P<0.05; throbbing = 2.1±0.6, P<0.01; sharp pain = 1.2±0.5, P<0.05; spreading = 2.4±0.7, P<0.01). For PHNTnc, reported sensations were more mild and fewer in number (i.e. aching = 1.0±0.3, P<0.01; dull pain = 0.8±0.3, P<0.05; sharp pain = 1.2±0.5, P<0.05; and spreading = 2.0±0.6, P<0.05). Significant differences between credible versus non-credible PHNT subgroups were noted for deep pressure (PHNTc = 2.2±0.6, PHNTnc = 0.6±0.2, P<0.05), heaviness (PHNTc = 2.3±0.7, PHNTnc = 0.6±0.3, P<0.05), fullness (PHNTc = 2.4±0.7, PHNTnc = 0.4±0.2, P<0.05), and numbness (PHNTc = 3.0±0.9, PHNTnc = 0.3±0.2, P<0.05) – all classic *deqi* sensations. Trending differences were noted for dull pain (PHNTc = 2.8±0.9, PHNTnc = 0.8±0.3, P = 0.06) and MI (PHNTc = 2.7±0.8, PHNTnc = 1.0±0.4, P = 0.08) (**Figure 4**).

### Acupuncture somatosensory stimulation effects: REAL vs PHNTc

**Figure 5:**
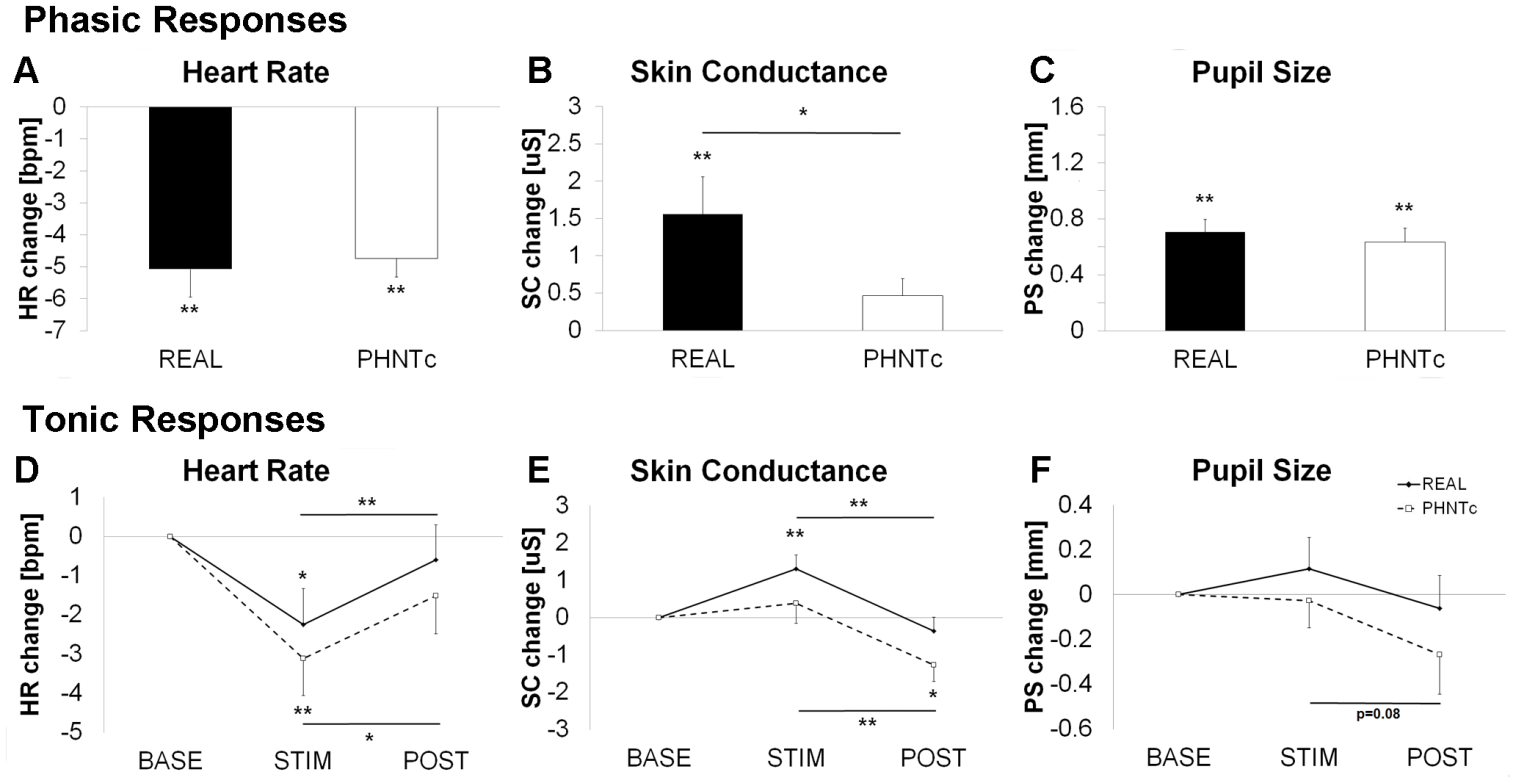
Influence of somatosensory needling on autonomic response modulation to real and phantom acupuncture. Phasic and tonic responses for heart rate (**A** and **D**), skin conductance (**B** and **E**), and pupil size (**C** and **F**) were contrasted between real (REAL) and credible (PHNTc) phantom acupuncture. Comparisons between REAL and PHNTc were based on the data collected only from subjects who regarded phantom acupuncture as real (i.e. PHNTc) and was done using paired *t*-tests. n.b. *<0.05, **<0.01. Error bars represent standard error of the mean.

Comparisons between REAL and PHNTc were based on the data collected only from subjects who regarded phantom acupuncture as real (i.e., PHNTc) and was done using paired *t*-tests. For phasic ANS response, there was significant phasic SC increase in response to somatosensory stimulation, for REAL (1.56±0.50 µS, P<0.001) but only trending response for PHNTc (0.47±0.23 µS, P = 0.07). We also noted significant phasic HR decreases (REAL = −5.07±0.87 BPM, P<0.001; PHNTc = −4.75±1.92 BPM, P<0.001) and PS increases (REAL = 0.70±0.10 mm, P<0.001; PHNTc = 0.64±0.10 mm, P<0.001) for both REAL and PHNTc sessions (**Figure 5A–C**).

We also noted significant tonic SC increase (STIM vs. BASE) for REAL (1.31±0.37 µS, P<0.01) but not PHNTc (0.39±0.54 µS, P = 0.49). Additionally, significant tonic SC decreases were noted from STIM to POST for both REAL (Δ = −1.67±1.15 µS, P<0.001) and PHNTc (Δ = −1.66±0.98 µS, P<0.001). For tonic HR response, significant decreases for STIM vs. BASE were noted for both REAL and PHNTc (PHNTc = −3.12±1.79 BPM, P<0.05; REAL = −2.24±1.35 BPM, P<0.01). Tonic HR rebounded back to baseline levels during POST (Δ = 1.60±0.98 BPM in PHNTc, P<0.05; Δ = 1.64±1.15 in REAL, P<0.001). Significant tonic PS decreases were found for POST (vs STIM) only in PHNTc (Δ = −0.31±0.66, P<0.05), but not in REAL (Δ = −0.24±0.48, P = 0.22) (**Figure 5D–F**).

**Figure 6:**
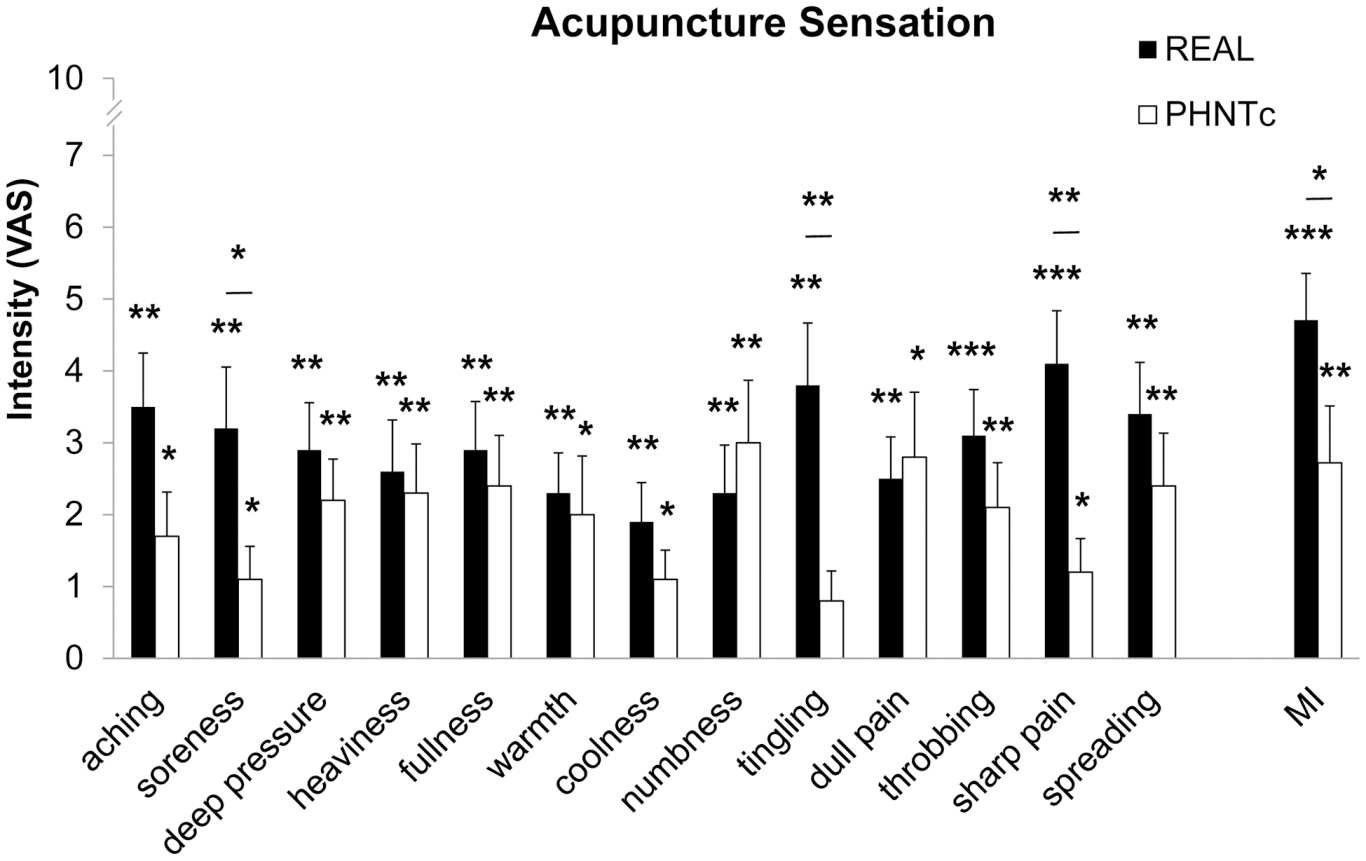
Influence of somatosensory needling on acupuncture sensations to real (REAL) and credible (PHNTc) phantom acupuncture. PHNTc reported similar sensation intensity as REAL for several *deqi*-related sensations (e.g., deep pressure, heaviness, fullness, numbness, dull pain). REAL produced greater sensation intensity for soreness, tingling, and sharp pain, as well as overall *deqi* sensation (i.e. MI). Comparisons between REAL and PHNTc were based on the data collected only from subjects who regarded phantom acupuncture as real (i.e. PHNTc) and was done using paired *t*-tests. n.b. *<0.05, **<0.01, ***<0.001. Error bars represent standard error of the mean.

Acupuncture sensations, such as aching, soreness, deep pressure, sharp pain etc., were reported following real acupuncture stimulation (REAL). Interestingly after PHNTc, even without any direct somatosensory stimulation, similar acupuncture sensation intensities as following REAL were also reported (aching pain: PHNTc = 1.7±0.6, REAL = 3.5±0.7, P = 0.07; deep pressure pain, PHNTc = 2.2±0.6, REAL = 2.9±0.7, P = 0.23; heaviness, PHNTc = 2.3±0.7, REAL = 2.6±0.7, P = 0.56; fullness, PHNTc = 2.4±0.7, REAL = 2.9±0.7, P = 0.43; numbness, PHNTc = 3.0±0.9, REAL = 2.3±0.7, P = 0.24; dull pain, PHNTc = 2.8±0.9, REAL = 2.5±0.6, P = 0.72). Greater sensation intensity was noted for REAL for a few other sensations, such as soreness (PHNTc = 1.1±0.5, REAL = 3.2±0.9, P<0.05), tingling pain (PHNTc = 0.8±0.4, REAL = 3.8±0.9, P<0.01) and sharp pain (PHNTc = 1.2±0.5, REAL = 4.1±0.7, P<0.01) (**Figure 6**).

Significant correlation between acupuncture sensation and autonomic response was found in PHNTc (deep pressure vs. phasic HR decrease: r = 0.73, P<0.05; deep pressure vs. phasic PS increase: r = −0.83, P<0.05; dull pain vs. phasic PS increase: r = −0.90, P<0.05) but not in REAL.

### Temporal evolution of ANS response to REAL and PHNT

**Figure 7:**
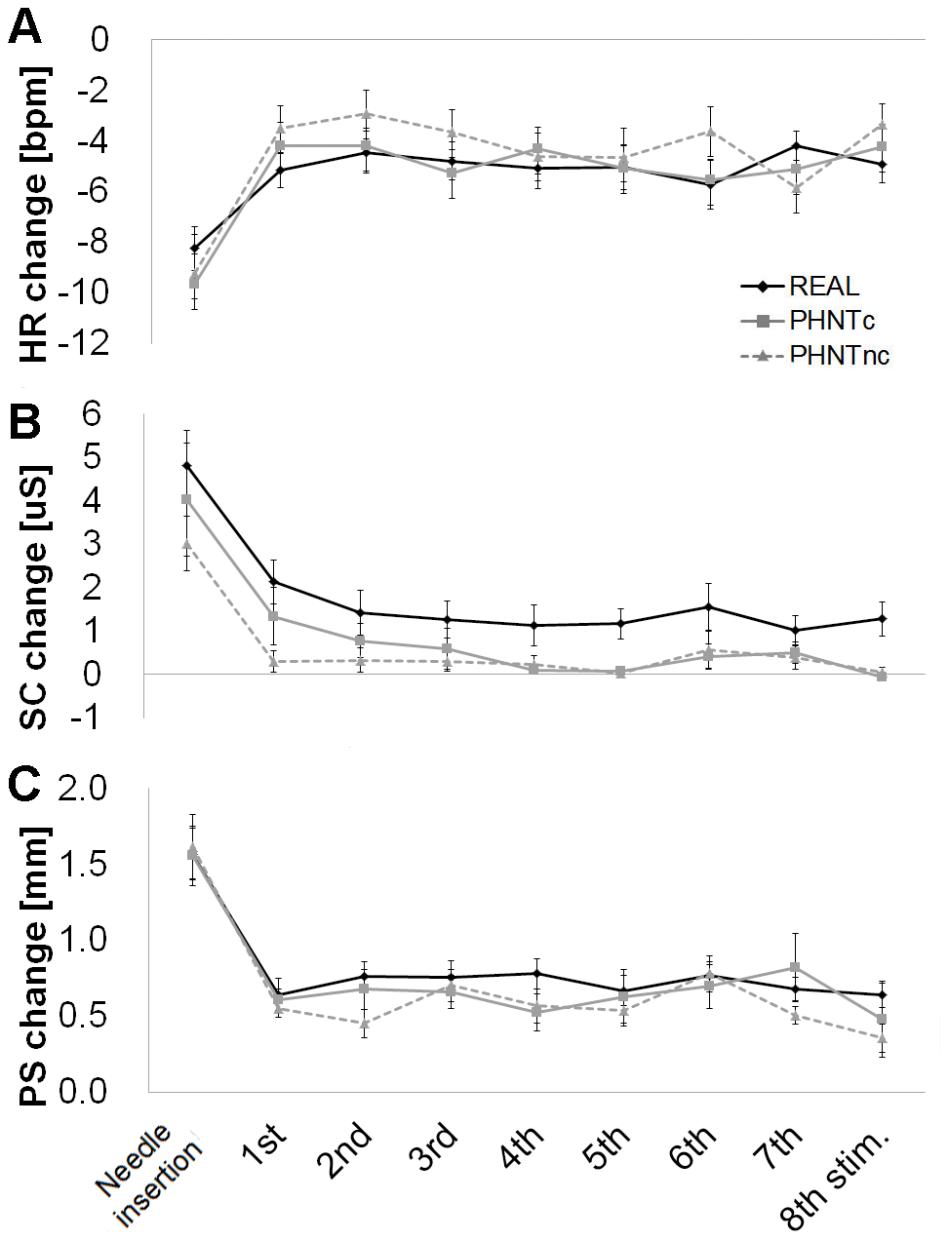
Temporal evolution of autonomic response to real (REAL) and phantom (credible, PHNTc; non-credible, PHNTnc) acupuncture. Needle insertion, whether real or phantom, produced significantly greater (A) HR decrease, (B) SC increase and (C) PS increase, compared to needle manipulation. ANS response to needle manipulation was relatively stable over all 8 manipulations for REAL (n = 20), PHNTc (n = 11), and PHNTnc (n = 9). SC increase was greater for REAL compared to PHNTc and especially PHNTnc, consistently over all stimuli. Error bars represent standard error of the mean.

During the needle insertion, before the needle manipulation (1^st^ –8^th^ stim.), significant HR deceleration was observed in the three groups (REAL = −8.25±3.85 BPM, P<0.0001; PHNTc = −9.67±4.02, P<0.0001; PHNTnc = −9.25±4.59, P<0.0001). The amplitude of HR decrease, in subsequent needle manipulation, was then reduced but still significant compared to the baseline (P<0.05 for all events, **Figure 7A** for individual response, **Figure 3A** for average response of eight stimuli). No significant difference was found between groups at each event.

Pupil size also showed maximum increase at needle insertion in all groups (REAL = 1.57±0.74 mm, P<0.0001; PHNTc = 1.56±0.62 mm, P<0.0001; PHNTnc = 1.62±0.63 mm, P<0.0001), and maintained greater than the baseline (P<0.01 for all events, except the response from PHNTc at 8^th^ stimulation; Δ = 0.48±0.79 mm, P = 0.09) (**Figure 7C** for individual response, **Figure 3C** for average response of eight stimuli). Significantly greater PS increase was observed at 8^th^ needle manipulation in REAL than PHNTc (REAL = 0.64±0.35 mm, PHNTc = 0.36±0.28, P<0.05) (**Figure 7C**).

Significant SC increase was also produced by needle insertion (REAL = 4.81±3.56 µS, P<0.0001; PHNTc = 4.02±4.30 µS, P<0.05; PHNTnc = 3.01±1.78 µS, P<0.001), and the responses were reduced in subsequent manipulation but significantly maintained compared to the baseline in REAL (P<0.05 for all event) but not in PHNTc (P>0.07 for all events) and PHNTnc (P>0.2 for all events) (**Figure 7B** for individual response, **Figure 3B** for average response of eight stimuli).

## Discussion

In this study, we have developed and tested a new form of placebo acupuncture, referred to as phantom acupuncture, which was characterized by an acupuncture needling intervention induced solely by visual display. We applied real (REAL) and phantom (PHNT) acupuncture and retrospectively re-classified subjects into two groups based on PHNT credibility (PHNTc, who found phantom acupuncture credible, n = 11; and PHNTnc, who did not find phantom acupuncture credible, n = 9). Physiological responses to REAL and PHNT were measured via autonomic response (heart rate, skin conductance, pupil size), while psychophysical responses were assessed by subjective ratings of needle sensation (Table 1). Real acupuncture induced greater skin conductance response, suggesting that the somatosensory component of acupuncture underlies the sympathetic outflow produced by acupuncture needle stimulation. We found that both real and phantom acupuncture (when credible) induced notable acupuncture sensation. The credibility of the ritual, a contextual component of acupuncture, was important for inducing robust *deqi* sensation, but was less important for autonomic response to purely visual phantom acupuncture, suggesting that some stimulus-associated autonomic response may be the result of sub-conscious processing that does not play a role in conscious cognitive re-evaluation of a ritual as credible or not.

**Table 1:** Summarization of the physiological responses to real and phantom acupuncture stimulation.

	Parasympathetic tone	Sympathetic tone	Orienting response	Deqi sensation
REAL		↑ (SC-phasic/tonic)	↓HR-phasic, ↑PS-phasic	+++ (for most sensation items)
PHNTc	↑ (SC-tonic, PS-tonic)		↓HR-phasic, ↑PS-phasic	++ (for deep pressure, heaviness, fullness,numbness, dull pain, and spreading)
PHNTnc			↓HR-phasic, ↑PS-phasic	+ (for dull and sharp pain)

HR: Heart Rate, SC: Skin Conductance, PS: Pupil Szie, tonic: tonic response, phasic: phasic response, +++: around 2–4 out of 10 scale, ++: around 2–3 out of 10 scale, +: around 1 out of 10 scale.

### Needling Credibility Effect: increased parasympathetic and decreased sympathetic activity

Physiological response to visual stimuli purporting to reflect needles entering the subject’s skin, and being twisted, depended on whether or not the subjects believed the procedure to be credible. By contrasting PHNTc with PHNTnc, we were able to explore the acupuncture needling context independent from any somatosensory afference. Our data demonstrated that while phasic autonomic response to visual scenes of needle stimulation were not influenced by needling credibility (i.e. PHNTnc vs PHNTc), tonic autonomic responses were influenced. Specifically, compared to PHNTnc, PHNTc demonstrated HR deceleration during the stimulation period and decreased SCR/PS following stimulation (see **Figure 3**). Needling credibility may be associated with greater positive expectation of acupuncture efficacy and a generally more relaxed state, reflected in increased parasympathetic and decreased sympathetic activity, consistent with our data.

Our results showed that needling credibility influenced multi-organ autonomic response to needle-related stimuli. Previous studies have found that several brain regions implicated in placebo responses, such as pregenual anterior cingulate cortex, amygdala, and periaqueductal gray [29] are also associated with peripheral autonomic outflow and are components of a central autonomic network [30]. Other studies have noted that expectancy enhances heart rate change and sympathetic responses to deep brain stimulation of subthalamic limbic region in Parkinson patients [31]. In addition, placebo analgesia has been linked with reduced beta-adrenergic (not cardiovagal) heart response [32], [33]. Thus, autonomic response may be an important factor in expectation and placebo-mediated outcomes. Phantom acupuncture clearly produces autonomic response and future studies should also link these multi-organ outflows with clinical outcomes in patient populations.

Interestingly, both phasic and tonic ANS response was evident for all three groups (REAL, PHNTc, PHNTnc), though more prominent in PHNT when the stimulus was judged to be credible. Visual feedback may be an important factor in augmenting a placebo intervention. Kaptchuk et al. have suggested that medical devices have an enhanced placebo effect and, specifically, that sham acupuncture is more effective than placebo pill on self-reported pain and symptom severity [34], [35]. This hypothesis was recently corroborated in a systematic review of migraine prophylaxis [36]. Most prior sham acupuncture procedures include tactile (in addition to visual) stimulation, and can be characterized by adequate needling credibility. However, tactile stimulation may be an important component of a specific acupuncture effect, and some researchers have raised this point in questioning previous efficacy clinical trials which included both real and sham control acupuncture procedures [11], [12]. While the visual component of acupuncture was found to also induce notable physiological response, particularly when phantom acupuncture was credible, our novel procedure was able to remove the somatosensory component and may be a viable procedure in future clinical trials aimed at dissociating the somatosensory versus visual components of acupuncture therapy.

Interestingly, the rated intensity of several acupuncture sensations (e.g. dull pain, heaviness, fullness, and numbness) associated with *deqi* sensation [27], were similar for PHNTc and REAL (see **Figure 6**). Hence, needling credibility leads to a mental rationalization of a perception anticipated by real needling (as all subjects experienced this at their initial session), even when a lack of somatosensory afference was incongruent to visual afference associated with needle insertion and stimulation. This effect may be similar to the rubber hand illusion, where body ownership is extended to an inanimate object, in this case a video recording [37]. Future studies should specifically explore if acupuncture sensation intensity can serve as a marker for needling credibility, and whether such sensations are closely linked to therapeutic efficacy in acupuncture trials. If sensation is the important variable for clinical outcomes, and if the sensation can be produced by needling credibility with no somatosensory afference, then this can be linked to the acupuncture placebo effect.

### Somatosensory stimulation effect: sympathetic activation in SC response

To investigate the somatosensory stimulation effect, REAL and PHNTc were compared. Both REAL and PHNTc included a visual feedback component and were both credible acupuncture interventions, though PHNTc did not involve somatosensory afference. REAL showed significantly greater phasic and tonic SC responses, while PHNTc did not demonstrate significant SC response. This suggests that sudomotor activity is specifically driven by the tactile component of acupuncture needle stimulation. Somatosensory afference can be delivered by acupuncture through an ascending pathway, which carries information from spinal cord to reticular formation, PAG and thalamus, and then to ACC, SI/SII, insula and prefrontal cortex, where tactile input can have broader cognitive/affective influence [7]. In our study, SCR was mainly observed with REAL stimulation, which suggests that somatosensory afference specifically supports the previously noted sympathetic response to acupuncture [5], [36] and may be similar to sympathetic modulation by other pain or pain-like stimuli [38].

### Visual stimulus effects: the orienting response

As previously noted, the visual stimulus itself may induce physiological response regardless of needling credibility. In fact, PHNTc, PHNTnc, and REAL all shared the same visual stimulus and produced physiological activity consistent with an orienting response (OR). Physiologically, OR is characterized by parasympathetically driven HR deceleration, sympathetically driven SCR increase, and behavioral orienting toward novel stimuli [39]. OR is also associated with pupil dilation linked to emotional processing of stimuli [40], [41]. These mixed autonomic responses likely reflect supra-spinal feedback and may be differentially associated with different aspects of cognitive and affective processing involved with attention distribution towards novel stimuli. Particularly for needle insertion, which was done before needle manipulation (see **Figure 7**), all three groups showed significant HR deceleration, SC increase, and PS dilation compared to the baseline, suggesting that the visual component of needle insertion (and perhaps needle manipulation) leads to a physiological OR. Subsequent needle manipulation events during the STIM period showed less robust ANS response compared to needle insertion, suggesting diminished salience to the subject leading to diminished physiological arousal. Importantly, lack of notable habituation across repeated needle manipulation stimuli for all three groups suggests that saliency was conserved and difference analyses using summary ANS outcome measures (pooled over all stimuli) were not confounded by preferential habituation in one or more groups.

The fact that all three groups demonstrated robust ANS response may have significant implications in terms of understanding the placebo effect. Most discussions of the placebo postulate that environmental learning cues mediated through either conscious expectations or classical conditioning are the principle psychological mechanism of placebo responses [42]. Recently there has been evidence that suggests that non-conscious and implicit framing may play a key role [43]. Our study found robust ANS outflow in response to phantom acupuncture, even when credibility was compromised. As sensation and autonomic response were likely to be classically conditioned from subjects’ experience in the training session with real acupuncture, any ANS outflow following phantom acupuncture, whether credible or not, may feed back to the brain via afferent autonomic pathways and play an important role in subsequent sub-conscious placebo effects.

### Psychophysical response to real and phantom acupuncture

Interestingly, while overall *deqi* sensation (i.e. MASS Index) was greater for REAL compared to PHNT, when the latter was credible, many key *deqi* sensations (e.g. dull pain, numbness, and deep pressure) were similar in intensity. This suggests that *deqi* sensations can be induced not only by somatosensory afference but also by visual suggestion of needle stimulation and needling credibility [37], [44]. In fact, when PHNT was credible, greater *deqi* sensation intensity (e.g. deep pressure) was associated with greater phasic HR decrease and with smaller phasic PS increase, suggesting that credibility-mediated acupuncture sensation raised parasympathetic activity, and the increased activity may be linked with clinical outcomes in patients (i.e. placebo effect) as the parasympathetic shift or the sympathetic drop has been believed to be one of underlying mechanisms in clinical acupuncture efficacy.

Several limitations should be noted. Our study was performed in healthy subjects (i.e., young university students) and not patient populations, and sample size of this study was quite small. As acupuncture is a therapeutic intervention applied for various pathological states, these results may not extend to, for instance, chronic pain patients. Thus, further study on large sample of patients should be performed for clinical implication. Additionally, our outcomes included autonomic outflow and psychometric outcomes. More clinically-relevant outcomes such as evoked pain modulation, should also be explored. Other physiological outcomes, such as brain response measured by neuroimaging should also be investigated.

In conclusion, our study developed and tested a new form of placebo acupuncture, referred to as phantom acupuncture, which was characterized by an acupuncture needling intervention induced solely by visual display. We found that both real and phantom acupuncture (when credible) induced notable acupuncture sensation. Real acupuncture induced greater skin conductance response, suggesting that the somatosensory component of acupuncture underlies the sympathetic outflow produced by acupuncture needle stimulation. We also found that credibility of the ritual, a contextual component of acupuncture, was important for inducing robust *deqi* sensation, but was less important for autonomic response to purely visual phantom acupuncture, suggesting that some stimulus-associated autonomic response may be the result of sub-conscious processing that does not play a role in conscious cognitive re-evaluation of a ritual as credible or not.
